# Supplementary material for: Combining 16S rRNA gene variable regions enables high-resolution microbial community profiling
Source: Microbiome. 2018 Jan 26;6:17. doi: 10.1186/s40168-017-0396-x (PMC5787238; doi:10.1186/s40168-017-0396-x)
Supplement: Additional file 1: — Supplementary Results. (DOCX 642 kb) [file 40168_2017_396_MOESM1_ESM.docx]

# Additional file 1

# Supplementary Results

## Theoretical effect of combining short regions

The *group* size of each bacterial sequence in the Greengenes (GG) database, namely the number of GG sequences that share the same sequence over the relevant amplified regions, was calculated. Figure S1 shows the fraction of GG sequences that belong to a *group* of up to a certain size, for one to six regions and for the V4 region. Using two regions was sufficient for better performance than V4. The sixth region seemed to provide only a small advantage over using the first five regions.


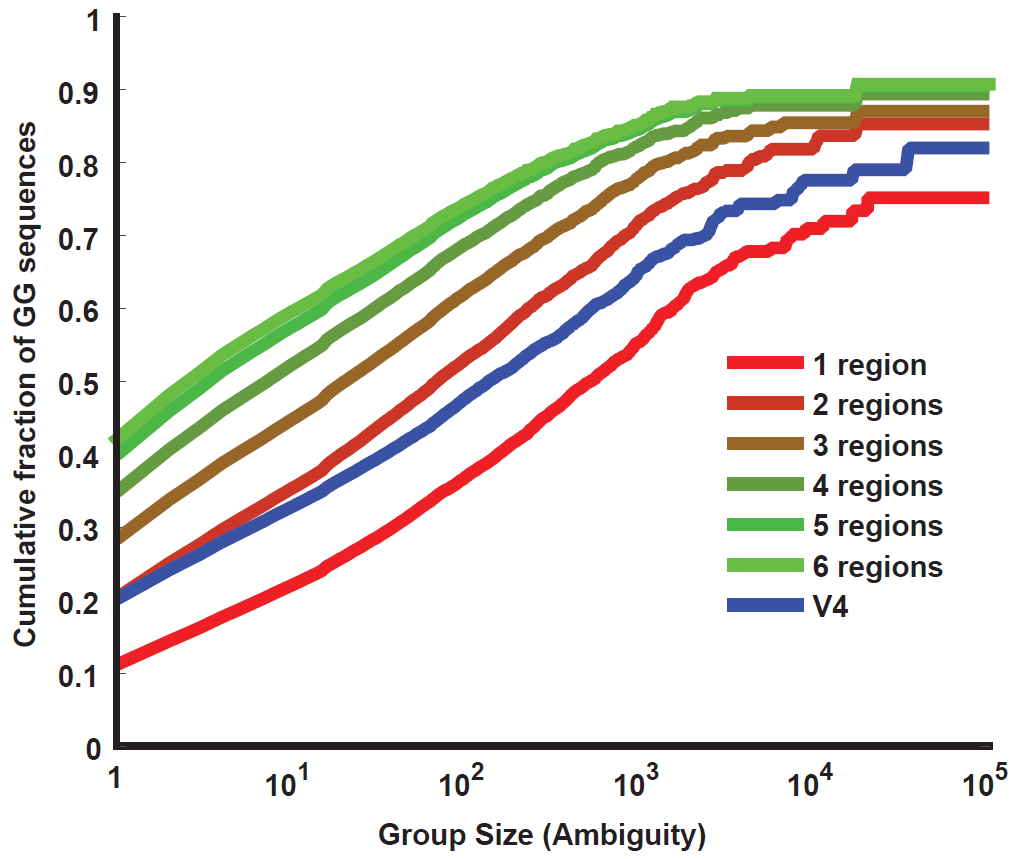


Additional file 1: Figure S1 (related to Figure 2A): Theoretical resolution. Each sequence in the Greengenes (GG) database was assigned to a '*group*', *i.e*., the set of GG sequences from which it is indistinguishable over the relevant amplified region(s). The figure shows the fraction of GG sequences that belong to a *group* of up to a certain size for one to six regions and for V4. The *group*'s size coincides with the *ambiguity* in this case and hence measures the resolution by which a bacterium may be identified.

***In silico* simulations**

A thousand mixtures were selected, each comprising one hundred bacteria randomly selected from the GG database and assigned a power law frequency distribution. Weighted precision and weighted recall were plotted for one to six regions and for using V4 primers, as a function of the *total* number of reads applied. The average number of reads per region was the total number of reads divided by the number of regions. Weighted precision monotonically improved when increasing the number of regions (two regions were sufficient to provide comparable precision to that of V4). Weighted recall was high in all cases, when the total number of reads was high enough (*e.g*., higher than 200,000). However, when allocating a low number of reads weighted recall decreased. This reduction in performance was more significant for higher number of regions, since the number of reads per region was insufficient to detect the low frequency bacteria.


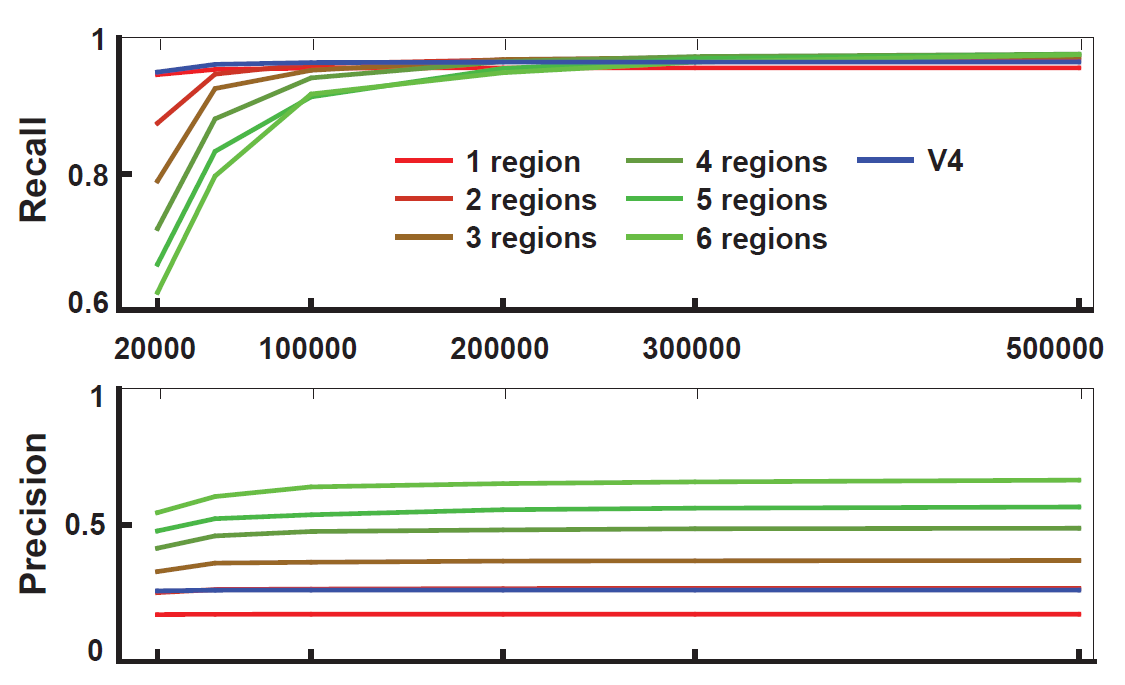


Additional file 1: Figure S2 (related to Figure 2B): Simulation results. Performance, *i.e*., weighted precision and weighted recall of simulated communities for one to six regions and for V4 as function of total number of reads. Error bars were omitted for clarity.

### Combinations of regions and their effect on mixture reconstruction

This section presents results across all possible subsets of regions up to six (as opposed to the former section that considered a single set of regions for each size). For each set of regions, 1000 mixtures were selected similarly to former simulations (using a total 200,000 reads across all regions). Figure S3 shows weighted recall, weighted precision and ambiguity across all sets of regions, segregated into singletons, pairs of regions, triplets, *etc*.. The 'size' of a set of regions refers to the number of combined regions.

*Weighted recall and precision*: Singleton regions displayed highly variable recall values, corresponding to the difference in universality among them. Weighted recall and precision for singleton regions were inferior to those of sets of regions. Weighted recall for all larger sets was high, while slightly decreasing for larger sets due to a lower number of reads per region. Weighted precision monotonically improved when increasing the number of regions. The variability in recall and precision among all combinations of regions of a certain size (*e.g*., among pairs) decreased with increasing the set size.

*Ambiguity*: Ambiguity was highly variable across different sets of regions, significantly decreasing when increasing the number of regions. Interestingly, ambiguity among sets of regions of the same size may vary by more than two fold. Although this phenomenon reflects the properties of the specific set of primer pairs, it shows that profiling would certainly benefit from a careful selection of sets of primers. In addition, in almost all cases it was favorable to use all possible primers rather than any subset of them.


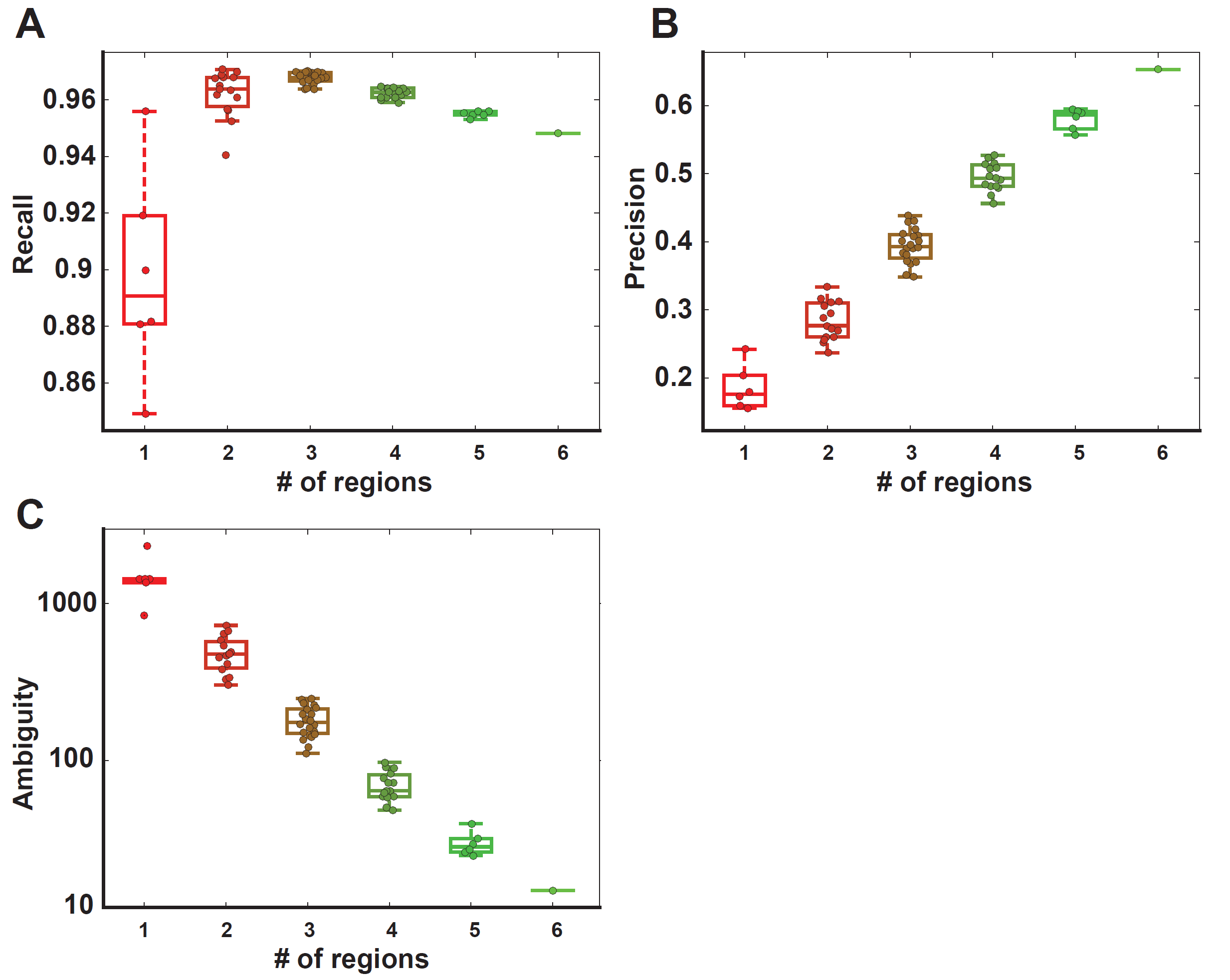


Additional file 1: Figure S3 Results across all subsets of regions. Performance, *i.e*., weighted recall (panel A), weighted precision (panel B) and ambiguity (panel C) of simulated communities for sets of one to six regions. Ambiguity is shown on a logarithmic scale.

### Read error effect

Performance was tested for a range of assumed constant $p_{e}$ values. For low values of $p_{e}$, the algorithm implicitly assumes that all reads are correct, and hence more bacteria appear in the reconstructed mixture and precision deteriorates. For higher values of $p_{e}$, the algorithm was able to correctly identify some of the noisy reads as such, which results in less falsely detected bacteria. Performance was robust for a wide range of $p_{e}$ values (Figure S4). Even under an extreme assumption of $p_{e}=0.001$ the degradation in precision was less than 3%. Based on these observations (and (Ross et al. 2013)) we chose to use $p_{e}=0.005$ for reconstruction of both simulated and experimental mixtures.


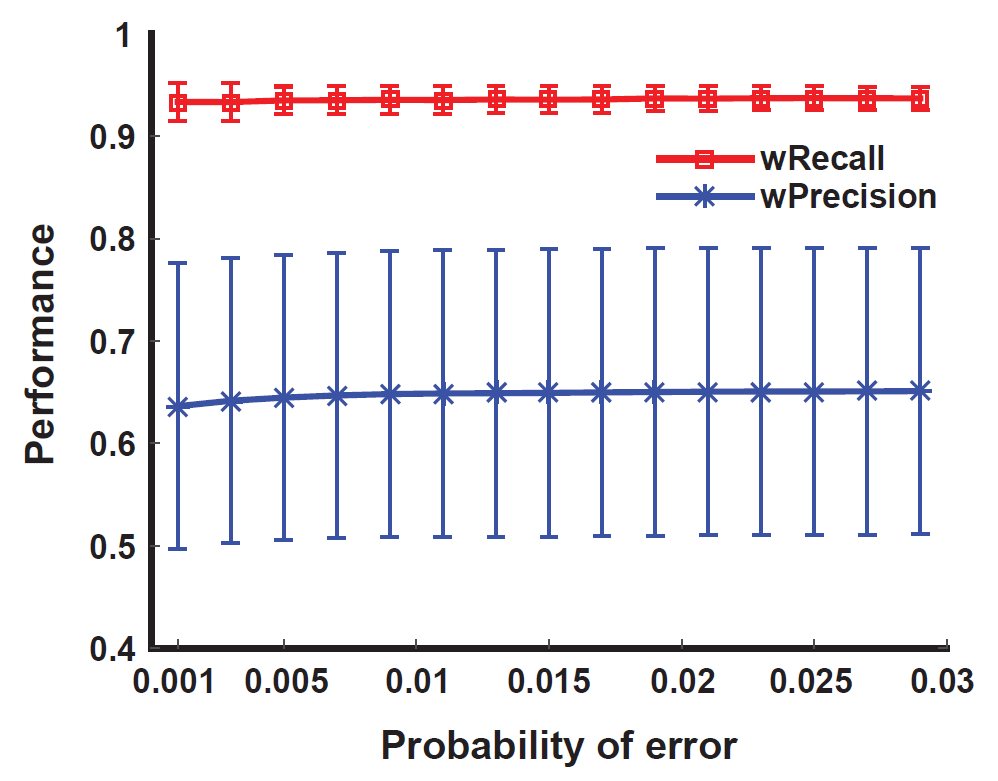


Additional file 1: **Figure S4: Weighted precision and recall averaged over 100 simulated communities as function of the value assumed for the sequencing error rate** $\boldsymbol{p}_{\boldsymbol{e}}$**.**

## Experimental mock mixture – normalizing the number of 16S rRNA gene operons

Table S1 compares reconstruction results based on six regions with and without adjusting for the number of 16S rRNA gene operons (operons for each bacterium were extracted by mapping the primers on its whole genome sequence). To compare results before and after normalization, the contaminations and false positive detections were excluded, and frequencies were renormalized to 1 in both cases. Operon adjusted reconstruction was closer to the original uniform distribution (Shannon diversity increased from 7.57 sequences to 8.94 sequences following operon adjustment), although for some bacteria the estimated frequency significantly deviated from the original proportion of 10%. This may be attributed to variable PCR amplification efficiency due to primer mismatches (the mixture was assembled using pre-extracted quantified DNA and hence extraction efficiency did not contribute to variable frequencies).

| **Mock mixture bacterial species** | # of operons | Not adjusted | Adjusted |
| --- | --- | --- | --- |
| *Escherichia coli* BL21 | 7 | 23.29 | 14.01 |
| *Lactobacillus casei* | 5 | 21.38 | 18.0 |
| *Bacteroides fragilis* | 6 | 14.02 | 9.84 |
| *Lactobacillus plantarum* | 5 | 11.49 | 9.67 |
| *Akkermansia muciniphila* | 3 | 10.26 | 14.4 |
| *Bacteroides caccae* | 5 | 9.06 | 7.63 |
| *Bacteroides ovatus* | 3 | 3.24 | 4.55 |
| *Eubacterium rectale* | 5 | 2.56 | 2.16 |
| *Eggerthella lenta* FAA 1-3-56 | 1 | 2.35 | 9.87 |
| *Eggerthella lenta DSM2243* | 1 | 2.35 | 9.87 |

Additional file 1: Table S1: Experimental mock mixture reconstruction results before and after operon correction. For each bacterium the number of operons identified in their whole genome sequence is shown together with the reconstructed frequency before and after adjustment for the number of operons, based on six regions.

## Experimental mock mixture

### Reconstruction accuracy

SMURF profiling of our experimental mock mixture was performed based on one to six regions. For most bacteria frequency seemed to 'stabilize' for two or more regions (Figure S5A). Also, the fraction of 'other' false positive detections decreased from ~2% to ~1% when using three or more region.

### Resolution as function of the number of regions

*Ambiguity* of species detected in one to six regions was measured by the exponent of Shannon's entropy (Figure S5B). Although all bacterial species were correctly detected for any number of regions and bacterial frequency seemed to stabilize even for two regions, in most cases resolution continued to improve as the number of profiled regions was increased. Due to inherent noise in the reconstruction process the resolution of a specific bacterium may not be strictly monotonic with increasing the number of regions. For example, the average group size of *B. ovatus* increased due to noise when we added the fourth region, but it improved again significantly when adding of the fifth region. An artificial example of such an effect is given below.


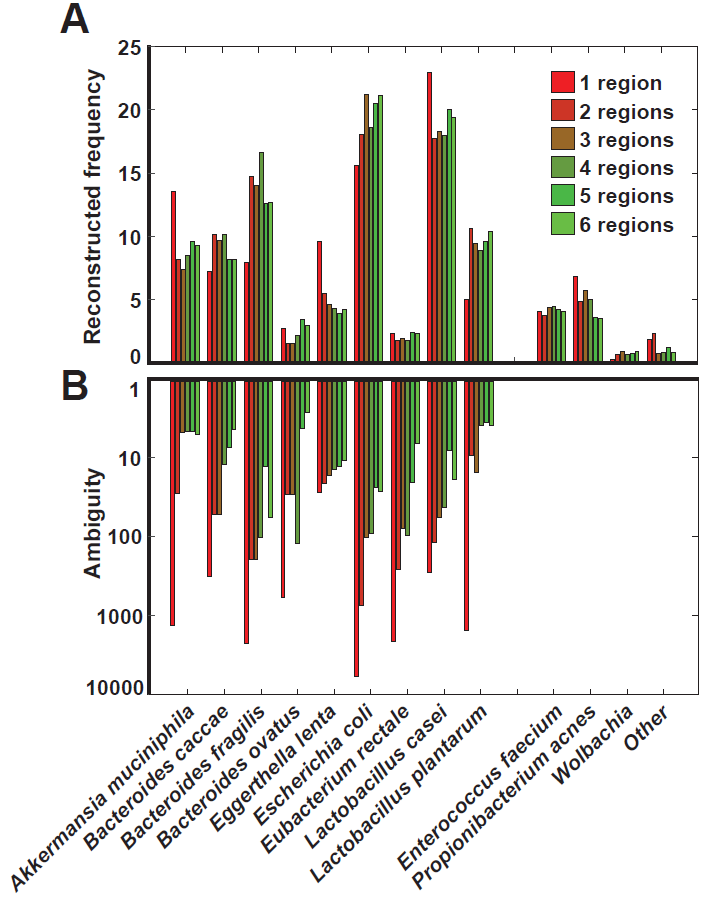


Additional file 1: Figure S5 (related to Figure 3) Experimental mock mixture. (A) Reconstruction results: Frequency (percent) of the reconstructed bacteria, both correct and false positive detections. (B) *Ambiguity* as a proxy for resolution: the exponent of Shannon's entropy of each bacterium in the mock mixture is shown, for one to six regions on a logarithmic scale.

### SMURF's results are robust to the selected 16S rRNA gene database

SMURF's reconstruction performed using the SILVA database^[[1]](#footnote-1)^ (Quast et al. 2013) was highly similar to the results obtained for the Greengenes-based SMURF (Figure S6). Results display robustness to the specific choice of sequences' database.


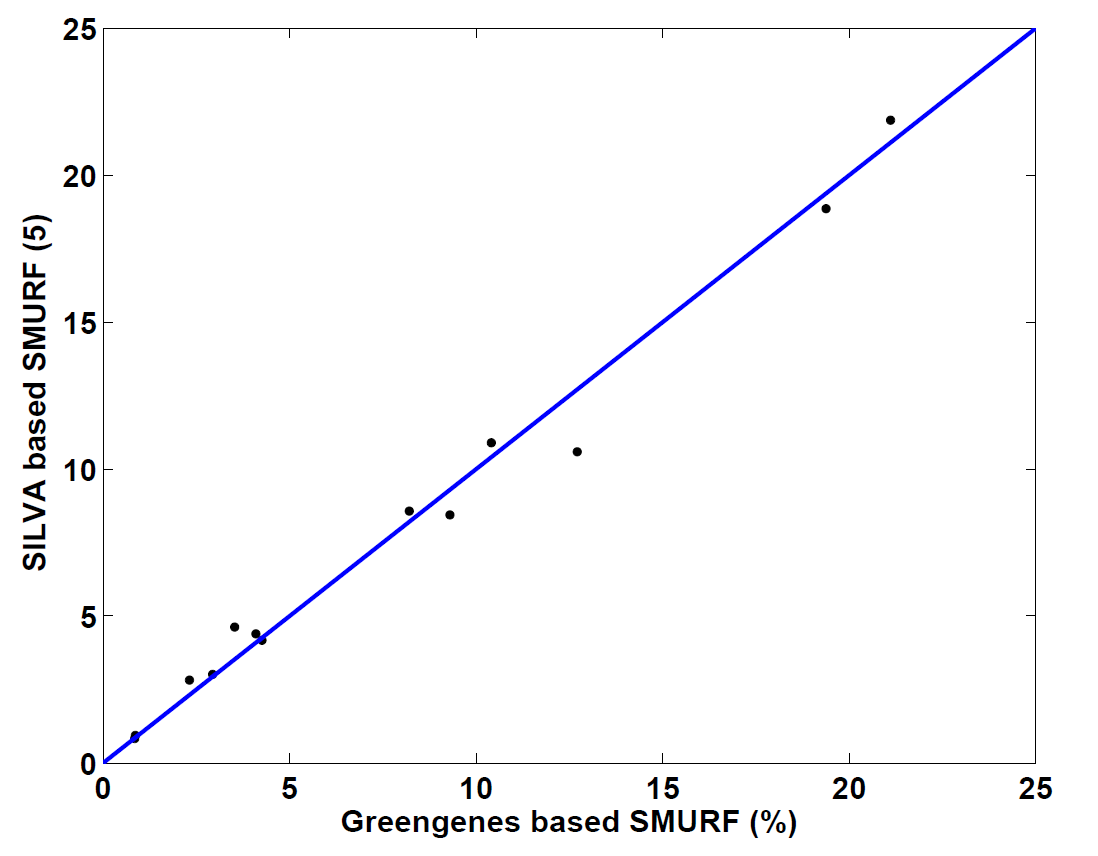


Additional file 1: Figure S6 Database dependence: A scatter plot of SMURF's mock mixture reconstruction (in percent) based on six regions: for a Greengenes-based SMURF (horizontal) vs. a SILVA-based SMURF (vertical). Each dot corresponds to a mock mixture bacterium.

### The effect of noise on resolution

The following synthetic example describes a scenario in which noise (*i.e*., PCR amplification errors and sequencing errors) causes a reduction in resolution when adding a region.

*The ad hoc database*: The 'experiment' is performed using two regions "1" and "2". The database includes a bacterium $A$ which is amplified by both primers' of region "1" and of region "2", and ten additional bacteria, $B,C,\ldots,K,L$, which are amplified only in region "2". The latter ten bacteria have the same sequence as bacterium $A$ in region "2".

Hence the matrix $M$ for a single region is:

$M=\begin{matrix} \begin{matrix} \begin{matrix} A & B & C \end{matrix} & \begin{matrix} D & E & F \end{matrix} & \begin{matrix} G & H & \begin{matrix} I & K & L \end{matrix} \end{matrix} \end{matrix} \\ [\begin{matrix} \begin{matrix} 1 & 0 & 0 \end{matrix} & \begin{matrix} 0 & 0 & 0 \end{matrix} & \begin{matrix} 0 & 0 & \begin{matrix} 0 & 0 & 0 \end{matrix} \end{matrix} \end{matrix}] \end{matrix}$

And for two regions, the matrix is:

$M=\begin{matrix} \begin{matrix} \begin{matrix} A & B & C \end{matrix} & \begin{matrix} D & E & F \end{matrix} & \begin{matrix} G & H & \begin{matrix} I & K & L \end{matrix} \end{matrix} \end{matrix} \\ \left[ \begin{matrix} \begin{matrix} \begin{matrix} 0.5 & 0 & 0 \end{matrix} & \begin{matrix} 0 & 0 & 0 \end{matrix} & \begin{matrix} 0 & 0 & \begin{matrix} 0 & 0 & 0 \end{matrix} \end{matrix} \end{matrix} \\ \begin{matrix} \begin{matrix} 0.5 & 1 & 1 \end{matrix} & \begin{matrix} 1 & 1 & 1 \end{matrix} & \begin{matrix} 1 & 1 & \begin{matrix} 1 & 1 & 1 \end{matrix} \end{matrix} \end{matrix} \end{matrix} \right] \end{matrix}$

In this synthetic example we assume that $p_{e}=0$ and thus$Q=M$.

*The 'mixture'*: Given the matrix $M$ we examine the results of profiling a 'mixture' containing a single bacterium $A$. Profiling is performed using region "1" and using both region "1" and region "2". We first consider the noiseless case and then introduce noise.

*'Experimental' measurement – the noiseless case*: In the absence of noise and since the mixture includes only bacterium $A$, the measurement vector $y$ for profiling based on only region "1" is given by $y=1$. The vector $y$ for profiling based on two regions is given by: $y=[\begin{matrix} 0.5 \\ 0.5 \end{matrix}]$.

The solution of the optimization problem for a single region and for two regions is the same in this case, and includes only bacterium *A*:

$$\boldsymbol{x}=[\begin{matrix} \begin{matrix} 1 & 0 & 0 \end{matrix} & \begin{matrix} 0 & 0 & 0 \end{matrix} & \begin{matrix} 0 & 0 & \begin{matrix} 0 & 0 & 0 \end{matrix} \end{matrix} \end{matrix}]$$

Resolution (*i.e*. *ambiguity*) in both cases is $1$.

*Experimental' measurement – the noisy case*: When profiling is based on a single region, noise has no effect since $y=1$.

Hence, $\boldsymbol{x}=\left[ \begin{matrix} \begin{matrix} 1 & 0 & 0 \end{matrix} & \begin{matrix} 0 & 0 & 0 \end{matrix} & \begin{matrix} 0 & 0 & \begin{matrix} 0 & 0 & 0 \end{matrix} \end{matrix} \end{matrix} \right]$ and *ambiguity* is $1$, as in the noiseless case.

However, assume that due to noise, the measurement vector for two regions slightly deviates from its noiseless value:

$$\boldsymbol{y}=[\begin{matrix} 0.45 \\ 0.55 \end{matrix}]$$

The solution of the optimization problem in this case would be

$$\boldsymbol{x}=[\begin{matrix} \begin{matrix} 0.9 & 0.01 & 0.01 \end{matrix} & \begin{matrix} 0.01 & 0.01 & 0.01 \end{matrix} & \begin{matrix} 0.01 & 0.01 & \begin{matrix} 0.01 & 0.01 & 0.01 \end{matrix} \end{matrix} \end{matrix}]$$

And hence the *ambiguity* is 1.74, which is larger than for a single region.

# Extended legends for manuscript's figures

Figure 1: A Schematic description of SMURF. (A) A schematic comparison between single and multiple region profilings. The two bacterial species (mocca and light gray) in the mixture are indistinguishable when profiling is based on a single (red) region. Moreover the existance of a third (light blue) bacterial species may not be ruled out. In contrast, when profiling is based on multiple regions (two in this case) the true composion may be reconstructed. (B) The SMURF flow diagram describing the internal steps of a typical analysis.

Figure 2: Theoretical resolution and in silico simulation results. (A) Theoretical resolution. The fraction of GG sequences that belong to a 'group' (*i.e*., the set of GG sequences that are indistinguishable over the relevant region) of up to a certain size for three cases: our single region, V4 and our six regions. Using six regions provided a significant increase in resolution, namely more sequences belonged to smaller groups, and hence may be better identified. The group's size coincides with the ambiguity in this case. (B) In silico simulation results. Weighted precision and weighted recall of simulated communities in three cases: six regions, a single region and V4. Error bars represent up/down mean absolute deviation over 1000 simulated communities.

Figure 3: Experimental mock mixture. (A) Reconstruction results: Frequency (percent) of correctly detected bacteria, and of false positive detections. Profiling was performed using a single region and using all six regions (legend applies to all panels). (B) Ambiguity as a measure of resolution: The exponent of Shannon's entropy of each bacterium in the mock mixture, shown for one and for six regions on a logarithmic scale. (C) The average ambiguity, which is ~100 fold smaller when using six regions compared to a single region.

Figure 4: Re-analysis of HMP data. (A) Reconstruction of the HMP 'Even' mock mixture (SRX 020130) from either V1-V3 or V6-V9 regions and from the two regions jointly (legend applies to all panels). (B) Ambiguity of profiling the HMP mock mixture: The exponent of Shannon's entropy of each bacterium in the HMP mock mixture SRX 020130, profiled either using V1-V3 or V6-V9 regions and by their combination.(C) Average ambiguity is reduced ~2 fold when combining the two regions.

**Figure 5. Reconstruction of bacterial populations in D. melanogaster following toxic treatment**. **(A)** Reconstruction of the bacterial populations at the species level in naïve flies using six regions (left) and one region (right). **(B)** Same for flies reared on medium containing the G418 toxin. Overall profiling at the species level was highly similar between one and six regions. **(C)** Ambiguity of the three most abundant species. Profiling based on a single region (hollow bars) resulted in much higher species ambiguity than for six regions (full bars), which allowed for much more efficient downstream analysis. **(D)** Similarity between *L*. *plantarum* predicted strains and the Sanger sequence of the strain isolated from the detected colony (the top 100 sequences are shown). The correct strain appeared in the predicted set for both one and six regions (dots and triangles, respectively), although the number of other L. plantarum false predictions were much larger in the case of a single region.

## References

Quast, C. et al., 2013. The SILVA ribosomal RNA gene database project: Improved data processing and web-based tools. *Nucleic Acids Research*, 41(D1).

Ross, M.G. et al., 2013. Characterizing and measuring bias in sequence data. *Genome biology*, 14(5), p.R51.

1. The SILVA version 128 resulted in a database of 2,014,374 16S rRNA gene sequences after accounting for sequence nucleotide ambiguities, and discarding sequences whose length was either longer than 2500bp or shorter than 1200bp. [↑](#footnote-ref-1)
